# Conspiracy Mentality: How it Relates to Populism, Relative Deprivation, Mistrust of Expertise and Voting Behaviour

**DOI:** 10.5964/ejop.10049

**Published:** 2024-02-29

**Authors:** Alexander Loziak, Dominika Havrillová

**Affiliations:** 1 Institute of Social Sciences of the Centre of Social and Psychological Sciences, Slovak Academy of Sciences, Košice, Slovakia; Dublin City University, Dublin, Ireland

**Keywords:** conspiracy mentality, populism, voting, populist attitudes, relative deprivation, mistrust of expertise

## Abstract

Background and research aims. Considering the high prevalence of conspiracy theories and misinformation, there is an urgent need to explain the tendency to adopt a conspiracy mentality and identify behavioural (including voting) outcomes of a high conspiracy mentality. The aims of the present paper are 1) the examination of populist attitudes dimensions, relative deprivation and mistrust of expertise as predictors of conspiracy mentality and 2) proposal of comprehensive models, that combine predictors of conspiracy mentality and its voting consequences. Methodology. Studies utilised OSL regression and structural equation modelling. Results. The overall regression was statistically significant. It was found that dimensions of populist attitudes (anti-elitism, sovereignty), relative deprivation and mistrust of expertise were significant predictors of conspiracy mentality. In line with the second research aim, the fitness of models was confirmed and results suggest mistrust of expertise is also a significant predictor of far-right voting. Discussion. The contribution of the paper lies in connecting conspiracy mentality with not only attitudes but also with important behaviour outcome - voting behaviour. We propose future research should experimentally examine whether the reduction of some of the identified predictors could possibly lower levels of conspiracy mentality and whether this reduction translates into voting behaviour.

International research on belief in conspiracy theories has seen a massive increase in recent years (Mancosu & Vassallo, 2022). The reason is an alarmingly high rate of spreading of false news and misinformation around the world, mostly through social media platforms (Cinelli et al. 2022). However, individuals differ not only in the extent to which they believe in conspiracy theories but also in their general susceptibility to explain phenomena and events based on these theories, which is called conspiracy mentality (Bruder et al., 2013). The political sphere is one of many areas significantly affected by conspiracy beliefs nowadays (Imhoff & Lamberty, 2018). Conspiracy theories are associated with the rhetoric of populist political leaders or with political orientation, exemplified by the positive relationship between conspiracy beliefs and right-wing parties. The belief in conspiracy theories also predicts human voting behaviour (Imhoff et al., 2022; Swami et al., 2018).

Despite the increasing attention to conspiracy beliefs, the construct of conspiracy mentality in psychological research is still relatively new and there is a lack of research examining its predictors and behavioural outcomes. It is important to study why some people hold certain opinions and which factors make people more prone to believe in conspiracy theories due to its negative consequences for personal and public life (e.g. rejecting scientific consensus, reducing intention to vote or the tendency to vote for radical parliamentary parties, etc.) (Mikušková, 2021). Existent research studies on conspiracy mentality are usually inconsistent, based on small samples and related variables are often examined in isolation (Farkhari et al., 2022). Next, the relationship between conspiracy mentality and populist attitudes has been explored, usually by looking at populist attitudes as a whole, not by their individual dimensions (Balta et al., 2022). There is also almost a complete absence of studies examining the relationship between conspiracy mentality and relative deprivation.

This study overcomes the gaps of existent conspiracy studies by providing a new and more comprehensive perspective on the relationship between conspiracy mentality, relative deprivation, mistrust of expertise, populist attitudes and voting behaviour using SEM analysis. It also contributes to the knowledge of predictors of conspiracy mentality in a representative sample of respondents. The variables of interest are studied in context with each other, not in isolation. Moreover, populist attitudes are explored by their dimensions, which provide more clear information about the concepts within populist attitudes that are related to conspiracy mentality.

The aims of the present study are 1) the examination of populist attitudes dimensions (anti-elitism, sovereignty, homogeneity), relative deprivation and mistrust of expertise as predictors of conspiracy mentality and 2) to propose comprehensive models that combine predictors of conspiracy mentality and its voting consequences.

## Background

### Conspiracy Mentality

Conspiracy theory, defined as the belief that several important social events, mostly negative, are the result of secret plans of powerful individuals, is a phenomenon that is commonly occurring in different times, populations and cultures (van Prooijen & van Vugt, 2018). Such theories can help people answer questions about why something happened, who should be blamed and who benefits from this situation. However, people differ in their predisposition to explain events as conspiracies. Some are more likely to believe in conspiracy theories than others, suggesting that belief in conspiracy theories can be considered as a trait-like predisposition, which is referred to as a „conspiracy mindset“ or „conspiracy mentality“ (Bruder et al., 2013).

The conspiracy mentality is a relatively stable personality trait describing individual differences in the extent to which people believe in conspiracy theories. Thus, it reflects the general tendency to believe in conspiracy theories (Imhoff & Bruder, 2014). There are three main motives that drive the belief in conspiracy theories (Douglas et al., 2017). According to epistemic motives, conspiracy theories provide simple explanations and help people understand the world in a situation where they face uncertainty. Second, existential motives are based on the fact that conspiracy theories can offer people a sense of security and safety in threatening situations. Third, social motives explain that belief in conspiracy theories can help people maintain a positive image of themselves in a group. Belief in conspiracy theories has a number of negative consequences for society. Among the most common are rejection of science, such as refusal of vaccination (Bertin et al., 2020) or negation of climate changes (van der Linden, 2015), hostility against outgroups and reduced confidence in government (van der Linden et al., 2021).

Currently, considering the relatively high prevalence of conspiracy theories, research efforts aim to explain the tendency to adopt a conspiracy mentality and identify its behavioural outcomes (e.g. Farkhari et al., 2022; Imhoff & Bruder, 2014). Researchers examine specific factors or predictors contributing to the belief in conspiracy theories, such as socio-demographic, psychological (personality traits, cognitive and social factors) or political factors. (Douglas et al., 2019). Although some previous studies examined conspiracy mentality as a predictor (e.g. Mikušková, 2021; Nera et al., 2022), we decided to approach it as a dependent variable following the prevalent line of research (e.g. Bertin et al., 2020; Landrum & Olshansky, 2019).

### Conspiracy Mentality and Feelings of Relative Deprivation

Feelings of relative deprivation are defined as the increased perception of one's own economic vulnerability. This concept reflects a person´s own vulnerability and disadvantaged position as a consequence of perceived injustice that also connects to perceived discrimination against ‘people like us’, who in society ‘never get what they deserve’ (Elchardus & Spruyt, 2016).

Recent research suggests relative deprivation (or related concepts) might have connections with conspiracy mentality. Salvador Casara and colleagues (2022) suggest that perceived economic inequality (which strongly conceptually relates to feelings of relative deprivation) is associated with greater conspiracy beliefs prevalence. This effect was fully mediated by the perception that society is breaking down (due to growing inequality), which increases conspiratorial thinking, likely in an attempt to recover a sense of order and control.

Explanation of the connections between conspiracy mentality and perception of economic vulnerability is quite simple—conspiracy beliefs promise to explain problems that economic vulnerability creates (Douglas et al., 2017). This vulnerability is also associated with a perceived lack of control, as feelings of relative deprivation are rooted in the perception of socially and economically disadvantaged positions. A recent meta-analysis (Stojanov & Halberstadt, 2020) confirmed lack of control is related to greater endorsement of conspiracy beliefs. Also, high levels of feelings of relative deprivation are dependent on the social comparison (I do not get what I deserve but others do) and because of it, the need to maintain a positive image of the self. Thus, believing in conspiracy theories might be a way to boost one's own image by feelings of importance and specialty, as well as to deal with unpleasant feelings and insecurities that might stem from relative deprivation (van Prooijen, 2022).

In line with these observations, we formulated the following hypothesis:

H1: Higher feelings of relative deprivation predict a higher conspiracy mentality.

### Conspiracy Mentality and Mistrust of Expertise

The next factor, mistrust of expertise, indicates a general scepticism of science and expert opinions. Mistrust of science, as a system of knowledge, often implies the suspicion of and lack of confidence in the independence of researchers and the scientific systems from large corporations (e.g. often pharmaceutical companies) as well as governments (Lamberty & Imhoff, 2018; Mari et al., 2022). This concept reflects the idea that the views of ordinary people are considered to be more valid than expert opinions (Oliver & Rahn, 2016).

In this sense, people can reject well-established scientific theories and can believe in fabricated, deceptive claims. A mistrusting mindset usually occurs in situations that create fear, insecurity or when one's own vulnerability is perceived. These situations can be associated with low self-esteem, poorer mental well-being, helplessness or anger (Landrum & Olshansky, 2019).

Researchers have examined contributors, which shape attitudes related to mistrust in science and experts. One of them is belief in conspiracy theories or conspiracy mentality. There is a large body of evidence showing a negative relationship between conspiracy theory beliefs and trust in science. For example, according to recent study (Nera et al., 2022), conspiracy mentality negatively predicted trust in scientific institutions during the pandemic of Covid-19 (e.g. the origin of the virus, vaccination against Covid-19). However, the effects of mistrust in science can also be seen beyond Covid-19. Other studies have suggested that low trust in science is associated with low acceptance of a wide range of scientific information, among the most famous are negation of climate changes (van der Linden, 2015) or genetically modified food (Scott et al., 2018). Scientific evidence is often rejected because they are seen as a product of conspiracy (Rutjens & Većkalov, 2022).

Based on these findings, we assume the following hypothesis:

H2: Higher mistrust of expertise predicts a higher conspiracy mentality.

### Conspiracy Mentality and Populist Attitudes

Populism, widely defined as an ideology that considers society to be divided into two homogenous and opposing groups, "the pure people" and "the corrupt elite", argues that politics should be following the general will of the people (Mudde, 2004). It is a multidimensional construct whose dimensions are defined in different ways by different authors. According to Schulz et al. (2018), the ideological core of populism is characterised by 3 key dimensions: anti-elitism, sovereignty and homogeneity of people.

A negative attitude towards elites, *anti-elitism*, describes the degree of dissatisfaction and distrust of people towards politicians. It expresses the rejection of a narrow group of politicians, who profit from power and pursue only their own interests. In this sense, elites are perceived as corrupt and deceiving people. The second dimension, the *preference for people's sovereignty,* highlights people as the central focus of politics. *People* in this context contain the majority, which is not *the elite*. This dimension represents the belief that the will and interests of people must be taken into account in important political decisions. As a last resort, it can be a matter of handing over power to the people, for example through a referendum vote. The third aspect, the belief in the *homogeneity of people*, represents the unity of ordinary people who share the same values, interests and way of thinking, whereas specific population segments are stigmatised as a threat or burden on society and are excluded.

Particularly, the strongest and significant relationship was found between anti-elitism, as the populistic dimension and belief in conspiracy theories. An explanation is that people who have negative stereotypes towards power holders, in combination with their experience of powerlessness are more likely to perceive conspiracy theories. This opposition between the powerless people and the powerful elites increases the likelihood of endorsing conspiracy theories (Christner, 2022).

Over the last few years, there has been a growing interest in researching the relationship between conspiracy beliefs and populism (Castanho Silva et al., 2017; Hameleers, 2021). Although populist attitudes relate more to the political aspect and conspiracy theories to society in general, their worldviews are very similar. Both populism and conspiracy theories try to reduce the complexity of world events by presenting simple stories of conspirators that control society and ordinary people as their victims. At the same time, their roots are in general hostility toward anything official (Castanho Silva et al., 2017). Several studies (e.g. Bergmann, 2018; Salvati et al., 2022) confirm the idea that populist voters are more likely to support conspiracy beliefs. Also, one of the correlates of populist attitudes is conspiratorial thinking (Erisen et al., 2021). The similarity between populism and conspiracy theories may lead us to expect that people who have developed populist attitudes tend to give greater credence to conspiracy theories.

In line with these research findings, we formulated the following hypothesis:

H3: Higher scores in populistic attitudes predict a higher conspiracy mentality.

### Conspiracy Mentality and Populist and Far-Right Voting Behaviour

The tendency to support conspiracy theories is also related to the political orientation of individuals. Several studies have found that people at the extreme ends of the political spectrum—at both left and right end are more likely than others to believe in conspiracy theories (Krouwel et al., 2017; van Prooijen et al., 2015). Although different types of people can engage in conspiracy thinking, according to research by Lamberty et al. (2018) people on the far right—conservatives and right-wingers—are particularly prone to believe in conspiracy theories compared to the far left. A recent study by Imhoff et al. (2022) in 26 countries has shown that the conspiracy mentality culminates in supporters of parties that are perceived as extreme right-wing and disapproving of liberal values. The explanation for this might be that people on the right are more likely to have personality predispositions that stimulate conspiracy thinking, such as low tolerance for uncertainty or a desire for simple solutions (van Prooijen et al., 2015).

Several studies suggest that conspiracy thinking can also influence voting behaviour (Swami et al., 2018). In the current political atmosphere, citizens/voters are confronted with fake news and alternative facts in the form of conspiracy theories. Populism is often characterised by a false discourse. It refers to conspiracy theories, questions and rejects the expertise and presents alternative truths that resonate better with common sense and the experience of ordinary people (Hameleers, 2020).

Some of the politicians of the populist parties, especially from the right-wing parties often use conspiracy rhetoric in their discourses. They actively contribute to the spread of conspiracy theories by providing false, harmful and unjustified explanations to influence people's beliefs or opinions and achieve particular social or political gain (Hameleers et al., 2018). Political conspiracies are typical for times of crisis which populist leaders can use in their favour (Moffitt, 2015). This populist communication is used as one effective tool to maintain the support of voters. Nowadays, social media also offers a great opportunity for populist leaders to spread their messages (Schneiker, 2019).

The spread of conspiracy theories by political leaders can be seen during the pandemic of Covid-19, accompanied by the denial of the danger of Covid-19 by far-right (and generally populist) politicians (Druckman et al., 2021). The pandemic represented the opportunity for populists to mobilise support and keep their popularity and political strength relatively stable. Such appeals are classic right-wing populist tactics (Magnus, 2022).

In line with these findings, we expect:

H4: Populistic attitudes, mistrust of expertise and relative deprivation have an effect on conspiracy mentality and conspiracy mentality has an effect on populist voting.

H5: Populistic attitudes, mistrust of expertise and relative deprivation have an effect on conspiracy mentality and conspiracy mentality has an effect on far-right voting.

## Method

### Participants and Procedure

A cross-sectional online sample representative of the Slovak adult population with respect to gender, age, education and region of residence was used and analysed in this research. Data were collected between 5th and 15th November 2021. Informed consent was obtained from all participants in the study. Participants were assured of confidentiality and anonymity of obtained data. From the initial sample (*N* = 902), 23 participants that completed the survey in less than 2 minutes were terminated. Data were also tested for careless responses by calculating longstrings and the Mahalanobis distance utilising R software. Using this method, 47 responders were removed from the sample. The final sample consisted of 832 participants (406 men, 426 women, average age *M* = 43.79, *SD* = 15.01). For all analyses, R software was utilised. Data, codebook, survey and R analysis script are available in Loziak and Havrillová (2023).

### Measures

The Slovak versions of each scale were provided using a forward translation procedure. Questionnaires are shortly described in this section and the full versions are available in *Full versions of scales* in Loziak and Havrillová (2023).

#### Dependent Variable Measurement

Conspiracy mentality was measured by *Conspiracy Mentality Questionnaire* (CMQ) developed by Bruder et al. (2013). It contains five items, e.g. “I think that events which superficially seem to lack a connection are often the result of secret activities”. Respondents answered the extent to which they think or do not think the following statements are true, on an 11-point scale, ranging from 0 (0% – certainly not) to 10 (100% – certain).

#### Predictors Measurement

Populist attitudes were measured by the scale of Schulz et al. (2018) consisting of twelve items divided into three dimensions: *anti-elitism* (4 items, e.g., “People like me have no influence on what the government does”), *sovereignty of people* (4 items, e.g., “The people, not the politicians, should make our most important policy decisions”), and *homogeneity of people* (4 items, e.g., “Ordinary people share same values and interests.”). The scale ranges from 1 (strongly disagree) to 7 (strongly agree).

Relative deprivation was measured using seven items from the scale of Elchardus and Spruyt (2016), e.g., “Government doesn’t do enough for people like me, others are always advantaged”. Respondents answered the degree of agreement with individual statements on a 5-point scale, ranging from 1 (strongly disagree) to 5 (strongly agree).

Mistrust of expertise was assessed by the questionnaire developed by Oliver and Rahn (2016), which contained three items, e.g., “When it comes to really important questions, scientific facts don’t help very much”. Respondents responded on a 7-point scale, ranging from 1 (strongly disagree) to 7 (strongly agree).

Voting behaviour was measured by two items: “Who did you vote for in the last parliamentary elections in 2020?” and “Who would you vote for in the next parliamentary elections?”. A list of political parties was provided to the respondents.

Regarding the first item, voters of 5 political parties (OĽANO, We are family, Homeland, SMER-SD and SNS) considered populist were classified as populist voters. Voters of the remaining 8 political parties and non-voters were classified as populist nonvoters. Voters of 3 political parties (ĽSNS, Homeland, We are family) considered far-right were classified as far-right voters. Voters of the remaining 10 political parties and nonvoters were classified as far-right nonvoters.

The classification (of populist and far-right parties) was made based on the relevant classifying system (Školkay et al., 2021). The classification was based on certain criteria—for example, demagoguery, nationalism or exclusion of certain groups.

Regarding the second item, participants who expressed an intention to vote for one of 5 political parties (OĽANO, We are family, SMER-SD, SNS and Voice) considered populist were classified as a group with the intention to vote populist party. Voters of the remaining 8 political parties and nonvoters were classified as a group without intention to vote populist party. Voters of four political parties (ĽSNS, Homeland, We are family, Republic) considered far-right were classified as a group with the intention to vote for a far-right party. Voters of the remaining nine political parties and non-voters were classified as a group without intention to vote for a far-right party.

### Descriptives and Correlation Analysis

Analyses were conducted using R software, packages jtools, devtools and performance. The descriptive statistics of all the used measures are reported in Table 1.

**Table 1:** Descriptive Statistics and Internal Consistency of Variables

Variable	*M*	Scale	*SD*	95% CI		Internal consistency	
				*LL*	*UL*	ω	α
conspiracy mentality	6.78	0-10	2.11	6.64	6.92	0.858	0.845
anti-elitism	5.97	1-7	1.10	5.90	6.05	0.748	0.733
sovereignty	5.60	1-7	1.26	5.52	5.69	0.843	0.839
homogeneity	3.96	1-7	1.40	3.86	4.05	0.822	0.817
relative deprivation	4.62	1-5	1.07	4.54	4.69	0.801	0.797
mistrust of expertise	3.73	1-7	1.24	3.65	3.82	0.637	0.601

The internal consistency of the used scales was adequate, with McDonald’s ω ranging from .637 to .858 (Cronbach’s α ranged from .601 to .845). We also report basic statistics for all conspiracy mentality items. In items “I think that many very important things happen in the world, which the public is never informed about” and “I think that politicians usually do not tell us the true motives for their decisions” recorded the highest means, suggesting participants were especially vulnerable to ideas of important issues being kept secret from the public. In Table 2, means, standard deviations, medians and modes for all conspiracy mentality items are available.

**Table 2:** Descriptive Statistics of Conspiracy Mentality Items

Item	*M*	*SD*	Md	Mo
I think that many very important things happen in the world, which the public is never informed about	7.86	2.40	9	10
I think that politicians usually do not tell us the true motives for their decisions	7.92	2.23	8	10
I think that government agencies closely monitor all citizens	4.90	3.05	5	5
I think that events which superficially seem to lack a connection are often the result of secret activities	6.54	2.72	7	5
I think that there are secret organizations that greatly influence political decisions	6.66	2.94	7	10

The mutual correlation of all the used variables are in Table 3. Conspiracy mentality positively and significantly correlated with anti-elitism, sovereignty, homogeneity, relative deprivation and mistrust of expertise.

**Table 3:** Correlations of Conspiracy Mentality With Other Variables

Variable	1	2	3	4	5
1. conspiracy mentality					
2. anti-elitism	0.44*				
3. sovereignty	0.51*	0.58*			
4. homogeneity	0.29*	0.13*	0.37*		
5. relative deprivation	0.52*	0.35*	0.43*	0.32*	
6. mistrust of expertise	0.41*	0.11*	0.29*	0.36*	0.43*

*p < .001.

The first aim of our study was the examination of populist attitudes dimensions including anti-elitism, sovereignty and homogeneity, along with relative deprivation and mistrust of expertise, as predictors of conspiracy mentality using regression analysis.

## Results

### Results of the First Research Aim

OSL regression was conducted to test if anti-elitism, sovereignty, homogeneity, relative deprivation and mistrust of expertise significantly predicted conspiracy mentality. Firstly, all required assumptions were tested to establish the adequacy of the data for regression analysis. All assumptions except for the assumption of homoscedasticity were met. Because the assumption of homoscedasticity was not met, robust standard errors instead of conventional SEs were utilised. Standardized regression coefficients mean-centred and scaled by 1 *SD* are reported. Regression statistics are available in Table 4.

**Table 4:** Summary of Linear Regression Analysis

Predictor	Est.	*SE*	*t*	*p*	Collinearity	
					Tolerance	VIF
Intercept	6.78	0.06	121.56	< .001		
anti-elitism	0.43	0.08	5.65	< .001	0.64	1.58
sovereignty	0.44	0.08	5.58	< .001	0.59	1.84
homogeneity	0.06	0.07	0.96	0.34	0.74	1.29
relative deprivation	0.55	0.08	7.22	< .001	0.69	1.47
mistrust of expertise	0.45	0.07	6.51	< .001	0.79	1.34

*Note*. Dependent variable: conspiracy mentality. *F*(5,826) = 123.87, *p* ≤ .001, *R*^2^ = 0.43, Adj. R^2^ = 0.43.

The overall regression was statistically significant, *R*^2^ = 0.43, *F*(4, 827) = 154.58, *p* = .001. It was found that all predictors except homogeneity (as a dimension of populistic attitudes) in regression significantly predicted conspiracy mentality.

The second aim of our study was to propose comprehensive models that combine predictors of conspiracy mentality and its voting consequences using SEM models. The models combine regression analysis concerning predictors of conspiracy mentality and regression analysis concerning conspiracy mentality as a predictor of voting behaviour. We have also explored other variables as possible predictors of voting behaviour. The advantage of this analysis is the mapping of relationships between variables with greater precision and comprehensiveness. SEM analysis also allows the evaluation of the overall fit of the suggested models.

### Results of the Second Research Aim

All the variables in regression analysis except for homogeneity (which was proved not to be a significant predictor of conspiracy mentality) were utilised in SEM analyses.

Analyses were conducted using R software, packages tidyverse, dplyr, ggpubr, and rstatix. Four comparisons in total were made. Compared groups, group sizes, means and standard deviations are reported in Table 5.

**Table 5:** N, Mean and SD of Conspiracy Mentality for Groups of Nonvoters and Voters

Group	*N*	*M*	*SD*
**populist vote**			
non-voter	430	6.53	2.21
voter	402	7.05	1.97
**far-right vote**			
non-voter	648	6.58	2.15
voter	184	7.49	1.79
**intention to vote populist**			
no intention	519	6.61	2.16
intention	313	7.05	2.01
**intention to vote far-right**			
no intention	685	6.65	2.12
intention	147	7.36	1.99

Means score for Conspiracy mentality scale is higher for voters of populist/far-right parties (compared with non-voters) and for groups with the intention to vote for populist/far-right parties (compared with groups with no intention).

**Figure 1:**
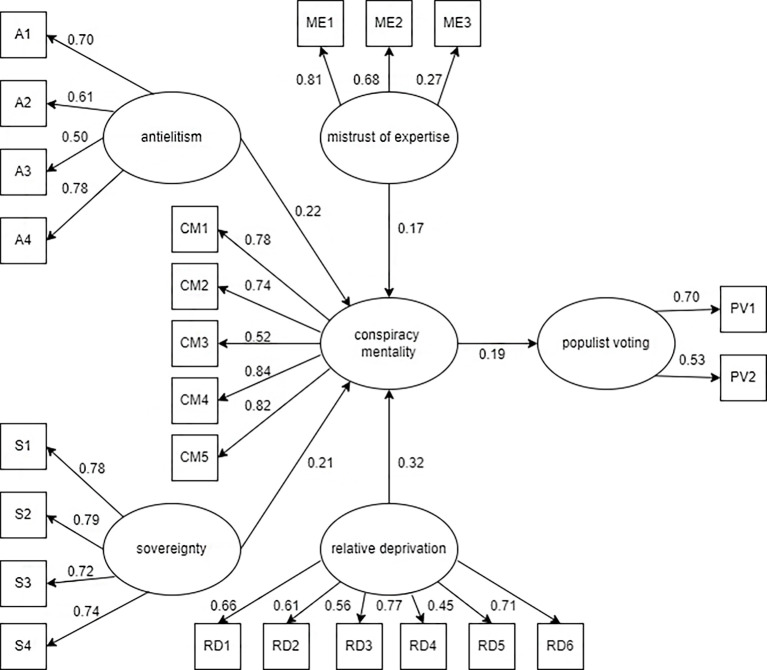
Model 1

Firstly, a normality assessment of items was conducted, using R software (lavaan, 2012). All items met recommended criteria of Brown (2006), with values of skewness falling between -3 and +3, and kurtosis falling between -10 to +10, when utilizing SEM. Then, the structural model of conspiracy mentality, antielitism, sovereignty, relative deprivation, mistrust of expertise and populist voting was developed. For purpose of this model, the latent variable 'populist voting' was created, consisting of actual populist vote and intention to vote populist in the next election. Calculations show model is overidentified. The path diagram of the model (Model 1) is shown in Figure 1.

All regression paths in the model are statistically significant (ranging from *p* < .000 to *p* < .001). The fit of the model is evaluated in Table 7.

**Table 7:** Evaluation of Fitness of a Structural Model

Category	Index	Level of Acceptance (*p*)	Index Value of Model 1	Index Value of Model 2	Index Value of Model 3
**Absolute Fit**	Chi-square	> .05	0.000	0.000	0.000
	RMSEA	< .07	0.051	0.051	0.050
	sRMR	> .05	0.050	0.052	0.050
**Incremental Fit**	CFI	> .90	0.931	0.931	0.934
	TLI	> .90	0.921	0.921	0.924

**Figure 2:**
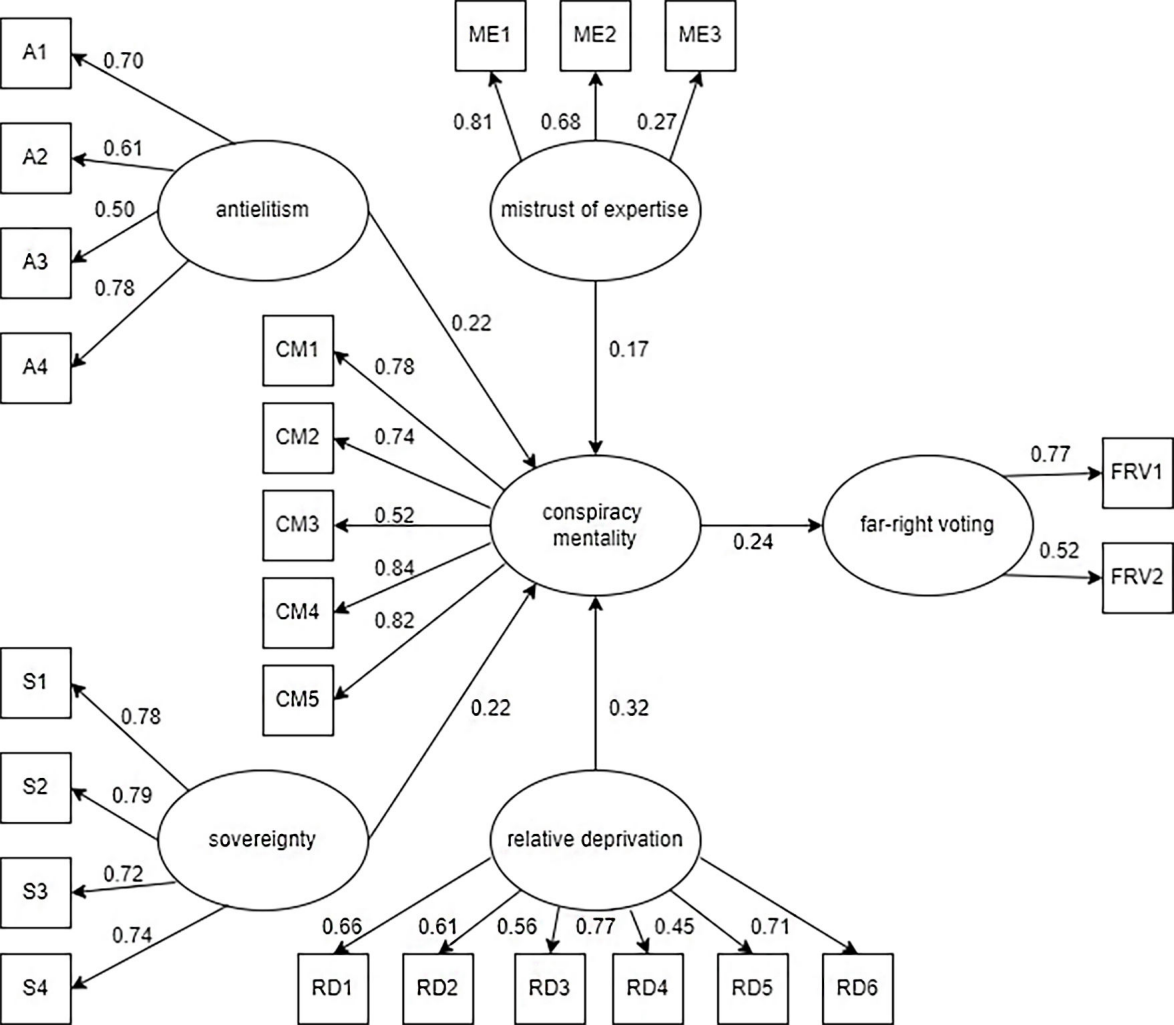
Model 2

A model predicting far-right voting (Model 2) was also developed, using the same structure of paths as in Model 1. The latent variable 'far-right voting' consisted of indicators of far-right vote and intention to vote far-right. The path diagram of Model 2 is shown in Figure 2.

All regression paths in the model are statistically significant (ranging from *p* < .000 to *p* < .001). The fit of the model is also evaluated in Table 7.

**Figure 3:**
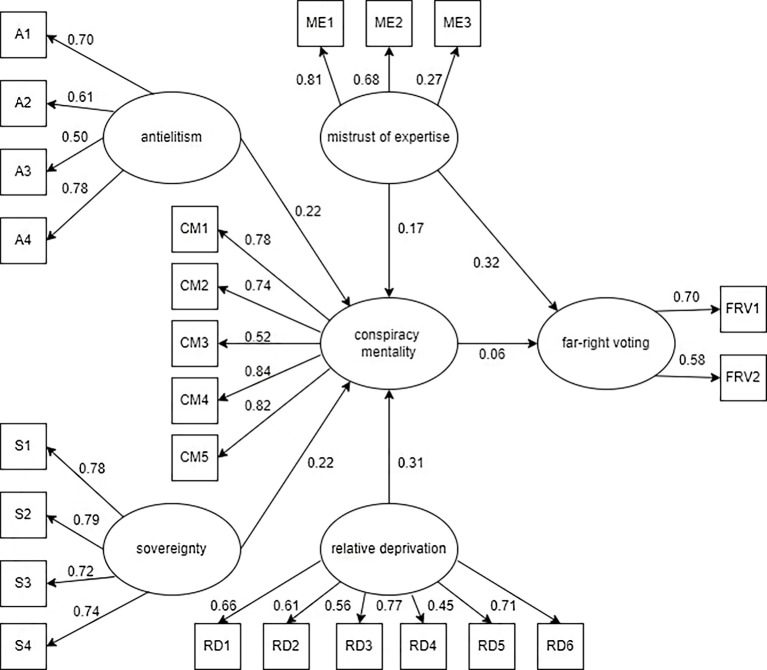
Model 3

Modification indices calculated in Model 2 revealed possible connections between variables of far-right voting and mistrust of expertise. Therefore we decided to add mistrust of expertise as a predictor of far-right voting in Model 3. The path diagram of Model 3 is shown in Figure 3.

Mistrust of expertise was confirmed to be significant (*p* < .000) and based on the value of standardised estimate (0.32) also a fairly strong predictor of far-right voting. However, the path between conspiracy mentality and far-right voting is no longer significant in this model (*p* < .294).

The fitness of models was evaluated by several indices, such as chi-square, RMSEA or CFI. Index values of indices of all models are available in Table 7.

Most of the values of calculated indices ranged within acceptable levels, indicating a good level of fit for all three models.

## Discussion

Present paper examined populist attitudes dimensions, relative deprivation and mistrust of expertise as potential predictors of conspiracy mentality. The regression including these predictors was statistically significant, with 43% of explained variance. Results revealed all predictors except homogeneity significantly predicted conspiracy mentality. The correlation matrix revealed positive weak (anti-elitism, homogeneity, mistrust of expertise) to positive moderate (sovereignty, relative deprivation) correlations between variables and conspiracy mentality. This confirmed H1, H2 and H3 of the present study. These results also confirm previous research findings by (Erisen et al., 2021; Hameleers, 2021; Nera et al., 2022).

It is interesting that anti-elitism and sovereignty were significant predictors of conspiracy mentality but homogeneity was not. It seems not all dimensions of populist attitudes have an equal relationship with conspiracy mentality. Although a degree of dissatisfaction towards politicians/government elites (antielitism) and belief that the will of people must be taken into account in political decisions (sovereignty) can predict conspiracy mentality, belief in the unity of ordinary people who share the same values (homogeneity) cannot predict it. A possible explanation of these findings may be found in the nature of conspiracy theories. One of the key aspects of conspiracy theories according to van Prooijen and van Vugt (2018) is an element of secrecy, which assumes 'the truth' is available to only a narrow special group of people. This implies the majority of the population is not aware of 'the truth' and because of it possibly does not share the same values as a special group of conspirators. The nature of conspiracy theories and the nature of homogeneity thus does not align with each other very well. Our results broaden other research findings that emphasise the role of anti-elitism in the relationship between populistic attitudes and conspiracy mentality (Christner, 2022).

A significant, positive and moderate relationship between feelings of relative deprivation and conspiracy mentality is the major finding of the present study. These findings are in line with previous research (Salvador Casara et al., 2022), supporting the idea that feelings of relative deprivation or perceived economic inequality in a population might create an environment in which conspiracy theories thrive. However, it is also possible, since we cannot determine the direction of causality of this relationship, that conspiracy mentality reinforces the perceptions of relative deprivation.

Proposed models supported H4 and H5. SEM analysis investigated relationships between variables in more specific, exact way and also explored connections of these variables to electoral behaviour. Conspiracy mentality was a significant predictor of both populist and far-right voting in the proposed models. These findings demonstrate how a higher tendency for conspiracy mentality directly affects the world around us, in this case, the political world. Although it is difficult to pinpoint the specific underlying mechanisms of studied relationships, some authors (Hameleers, 2020; Imhoff et al., 2022) do offer some explanation. Populist and far-right politicians often intentionally exploit conspiracy theories in pre-election campaigns, with the goal of gaining popularity with voters that believe in such theories. Populist and far-right political parties thus effectively mobilise this group of voters, which can be a significant portion of the population—during the Covid-19 pandemic as much as 49,7% of the North American population believed at least one conspiracy theory (Leibovitz et al., 2021). This could explain why voters of populist or far-right parties score higher in conspiracy mentality compared to non-voters. However, the exact causality of this relationship is unclear—we cannot assess the extent to which a conspiratorial mentality drives the election of these parties and, conversely, the extent to which political orientation fuels vulnerability to a conspiratorial mentality. Either way, this relationship can work both ways. Efforts need to be made to reduce the level of conspiratorial mentality and at the same time to increase critical thinking within this group. Such efforts could possibly reduce the success of populist or extremist political parties.

The results also revealed surprising findings regarding the mistrust of experts. Model 3 demonstrated that mistrust of experts is a better predictor of voting for the far-right than a conspiracy mentality. However, this is not the case for voting for populist parties. Thus, there is reason to believe that there are substantial differences in the predictors of populist and far-right voting. However, these findings need to be verified by further research.

One of the strengths of the paper is the sample size and its representativeness with respect to a number of characteristics. It is permissible to assume that the research results are generalizable to the population.

One of the contributions of the research is the comprehensiveness of the examination of the relationships that was undertaken in model testing. Our proposed models incorporate many variables relevant to current research and reveal important findings—for instance, the dominant role of distrust of experts as a predictor of voting for the far right.

Our study also has some limitations. Although it has some predictive capability, it cannot make conclusions on causality. An experimental study should be conducted to examine whether the reduction of the identified predictors could possibly lower levels of conspiracy mentality.

Considering alarming levels of conspiracy mentality in the population, conducting research on this topic is more important than ever. Conspiracy mentality has in recent years impacted crucial spheres of our society (public health, political behaviour) and there is no evidence this trend is weakening. Therefore, research needs to fully understand the concept of conspiracy mentality and identify tools that will be able to limit it.

## Supplementary Materials

For this article, data, codebook, survey and R analysis script are available (see Loziak & Havrillová, 2023).



LoziakA.
HavrillováD.
 (2023). Conspiracy mentality: How it relates to populism, relative deprivation, mistrust of expertise and voting behaviour
[Data, codebook, survey, script]. PsychOpen. https://osf.io/wcuq7
10.5964/ejop.10049PMC1093666538487597

## Data Availability

For this article, data is freely available (see Loziak & Havrillová, 2023).
